# Dietary patterns and nutritional status of HIV-infected children and adolescents in El Salvador: A cross-sectional study

**DOI:** 10.1371/journal.pone.0196380

**Published:** 2018-05-15

**Authors:** Rocio Martín-Cañavate, Michela Sonego, Maria Jose Sagrado, Gustavo Escobar, Estefanie Rivas, Sandra Ayala, Luis Castaneda, Pilar Aparicio, Estefania Custodio

**Affiliations:** 1 National Center of Tropical Medicine, Institute of Health Carlos III, Madrid, Spain; 2 Consortium for Biomedical Research in Epidemiology and Public Health (CIBERESP) Madrid, Spain; 3 National Center for Epidemiology, Institute of Health Carlos III, Madrid, Spain; 4 Field Epidemiology Training Program, National Center of Epidemiology, Institute of Health Carlos III, Madrid, Spain; 5 Centro de Excelencia para Niños con Inmunodeficiencias, Children Hospital Benjamin Bloom, San Salvador, El Salvador; 6 European Commission Joint Research Centre, Ispra, Italy; TNO, NETHERLANDS

## Abstract

**Introduction:**

The present study aimed to assess the nutritional status, the dietary patterns and its associated factors in the HIV-infected population of children and adolescents on antiretroviral treatment at the El Salvador reference center for pediatric HIV care (CENID).

**Methods:**

A cross-sectional survey was carried out between December 2010 and December 2011. Socio-demographic and clinical characteristics were collected from 307 children and adolescents aged 2–18 years and receiving antiretroviral therapy. Nutritional status was assessed by height-for-age, weight-for-height and body mass index-for-age. Dietary data was collected through a 24 hour recall, and through a weekly food frequency questionnaire. Dietary patterns were identified by principal component analysis. Bivariate and multivariable statistical methods were used to assess the factors associated with “high adherence” to the “healthy diet” pattern.

**Results:**

More than a third of the study group (33.2%) were stunted, 3.3% were identified as being wasted, and 10% were overweight or obese. Their diets were predominantly based on a high consumption of cereals, beans, eggs and processed foods and a low consumption of fruits, vegetables and dairy products. Three dietary patterns were identified: “healthy diet”, “high fat/sugar diet” and “low diversity diet”. Being female (OR: 1.63; 95%CI: 0.97–2.75), younger (OR: 2.37; 95%CI: 1.28–4.36) and institutionalized (OR: 14.5; 95%CI: 5.35–39.50) increased the odds to adhere to the “healthy diet” pattern.

**Conclusion:**

Our findings reveal a high prevalence of stunting and overweight in HIV-infected children in El Salvador. Institutionalized children were more likely to adhere to a healthy dietary pattern whereas children in poverty were more likely to have less varied and healthy diets. These results highlight the need to assess the dietary patterns of HIV-infected children and adolescents in order to guide public policies to design healthy life style interventions for this population at risk.

## Introduction

According to the 2015 UNAIDS report, about 20 000 people live with HIV in El Salvador, of whom less than 500 are children under the age of 15 years [1].

HIV-infection and malnutrition are strongly linked.For many years, the most common malnutrition form observed among HIV-infected children and adolescents worldwide was the acute malnutrition or wasting syndrome, characterised by significant decrease in body fat, and lean and bone mass, that was also accompanied by growth retardation or stunting [2] [3] [4].

After the introduction of the highly active antiretroviral therapy (HAART) in the nineties, the mortality and morbidity associated with HIV infection among children and adolescents was drastically reduced, and there was a significant improvement in the HIV-related wasting syndrome. On the other hand, children’s growth retardation associated with the infection became prevalent [5]. Moreover, HAART has been associated with metabolic alterations involving hyperglycaemia and dyslipidemia, as well as abnormalities in body fat distribution [6]. These alterations can compromise the health status of HIV-infected children and adolescents during their adult life resulting in an increased risk of cardiovascular disease, kidney diseases, liver diseases and osteopenia/osteoporosis [7] [8]. Two of the main factors for maintaining an optimal nutritional status and prevent these complications are diet and physical exercise.

Diet plays a crucial role in the immune system of HIV/AIDS patients, as sufficient amounts of macro- and micronutrients are essential for its normal functioning [9], whereas practising physical activity has been found to improve the lipid levels in HIV-infected children on ART treatment [10]. Therefore, the adherence to a healthy diet from the early to advanced stages of HIV infection in this population is essential in order to maintain a good nutritional status, to optimise health outcomes and to prevent future chronic complications [11] [12] [13].

El Salvador, as other countries in the region, is experiencing a nutrition transition by which diets based on local staples are giving way to rising consumption of foods high in fat, salt and, sugar and animal products [14] [15]. The consequences of this dietary changes can negatively impact the well-being of the HIV infected children and adolescents, as they are associated with increasing obesity rates, diabetes and other non-communicable diseases [16] [17].

However, although the exercise patterns have been previously described for this population [10] there was no study evaluating the dietary patterns and nutritional status. Therefore, we aimed to assess the nutritional status, the dietary patterns and its associated factors in all the HIV-infected children and adolescents diagnosed and treated in El Salvador.

## Materials and methods

### Study design and participants

This was a descriptive and analytical cross-sectional study of the population attended at the Centro de Excelencia para Niños con Inmunodeficiencia (CENID) in the Hospital Nacional de Niños Benjamín Bloom, San Salvador, where the total number of children diagnosed with HIV resident in El Salvador were receiving medical treatment at the time of the study.

### Data collection

During one year period, (December 2010 to December 2011), a survey was performed at one of the follow-up visits on all HIV-infected children monitored at the CENID (312 children). Informed written consent from parents or guardians of all children was obtained, as well as informed written assent from children twelve years or older. In the present study, we focused on the 307 children aged 2 to 18 years who were under antiretroviral therapy at the time of the survey, as children younger than 2 years may be on breastfeeding or weaning feeding periods, which could difficult dietary interpretations.

Information on socio-demographic and clinical characteristics was extracted from individual medical and social records. All children admitted at the CENID were classified by the social worker according to their socioeconomic conditions in one of these following six categories: 1) extremely poor, 2) poor, 3) low class, 4) medium class, 5) high class, or 6) “institution”, which included all the children living in institutions, either permanently or on week-days. The categories were assessed through the collection of information with a questionnaire including the characteristics of the child’s main caregiver (family tie to the child, educational level, type of work and health status) and of the household (economic income of all household members, type of dwelling and geographic area in which the household was located). Children living in institutions were mostly orphans whose parents had died from HIV or whose caregivers were not able to comply with the child’s antiretroviral treatment. We deleted the high class category due to no records reported, and collapsed the low and middle income categories due to the low number of children ranked in each of them. Therefore, the final socioeconomic adjustment variable contained only four categories: 1) extremely poor, 2) poor, 3) low-middle class and 4) institution.

At the time of the survey, the children were weighted and measured with a combined weighting scale and stadiometer (DETECTO®, model 43). Related indexes of the body mass index (BMI) and height-for-age z-scores were calculated for each child using the ANTHRO 1.02 software program (Division of Nutrition, CDC, Atlanta, GA) based on WHO growth reference values [18]. Wasting was defined as weight-for-height less than -2 standard deviations (SD) of the reference population in children under 5 years of age, or BMI-for-age z-score below -2SD in children equal or above 5 years. Stunting was defined as height-for-age z-score below -2SD, overweight as BMI-for-age z-score >+1SD and obesity as BMI-for-age z-score >+2SD. A variable combining both overweight and obesity was created, as there were few children with obesity in the sample.

Dietary data was collected through a 24 hour recall, and through a weekly food frequency questionnaire (FFQ). The food frequency questionnaire was self-elaborated based on available literature and on the results of the 24 hour recall collected during the pilot phase. The structure of the FFQ was based upon the 2006 version of the FFQ used in the Spanish National Health Surveys [19]. The food list was adapted to the El Salvador context based on the consultation of bibliography, nutrition local experts’ inputs, and the results of a pilot 24-hour recall conducted previously in the population of study. The food consumption questionnaires were administered to the children and their caretakers (only to caretakers in the case of children below ten years), and they were piloted before the roll-out of the study.

The frequency of intake was measured across five categories on the FFQ ranging from “Rarely or never” to “Daily”. The period used as a reference was a usual week. No information on the portion size of foods consumed was obtained.

### Statistical analysis

Frequencies and percentages were used to summarize data.

Dietary patterns were identified by principal component analysis (PCA) [20] using the frequency of consumption of the food groups in number of days a week, based on the weekly FFQ.

The food groups were entered into the PCA procedure using the software SPSS (SPSS; version 18.0), and nine factor solutions were obtained. We used the Kaiser criterion to identify the key dietary patterns by keeping only the factors with eigenvalues >1.0. The identified factors were orthogonally rotated to simplify the factor structure and to enhance their interpretability. Factor scores were calculated for each child by summing the products of the observed consumption frequency and the factor loading for each food group, in each of the food patterns identified. For interpretability, the food groups with factor loadings of |>0.2| were considered to contribute significantly to the pattern [21] [22]) and factors were labelled according to them.

The factor scores are continuous variables that were considered as indexes of adherence to the dietary pattern with which they were identified. They were subsequently divided in tertiles and dichotomized in a “high adherence” variable computed as 1 if the factor score was in the highest tertile and as 0 if it was in any of the lower tertiles, for each of the dietary patterns respectively.

Bivariate analyses using Chi-squared test were used to assess the variables associated with the “high adherence” of each of the dietary patterns identified. The variables tested were the following: age, sex, socioeconomic status, rural/urban residence, child’s caregiver/tutor, viral load and time of treatment. All variables associated with a p-value<0.1 in at least one of the the bivariate analysis were initially included in the construction of the multivariable models. Logistic regression models were obtained by using a manual backward stepwise procedure. P-values less than or equal to 0.05 were considered statistically significant. The unadjusted and adjusted odds ratio (uOR and aOR, respectively) with the 95% confidence intervals (95% CI) were computed. Bivariate analysis using Chi-squared were also used to assess the association between nutritional status and dietary patterns. STATA 13.0 (College Station, TX: StataCorp LP) was used for the construction of the models and the bivariate analysis.

### Ethical considerations

Informed written consent was obtained from parents or guardians of all participant children. In addition, informed written assent was obtained from children that were 12 years or older at the time of the study. The study was approved by the Clinical Research Ethical Committee (Comité de Etica en Investigacion Clinica) of the Hospital Nacional de Niños Benjamín Bloom in El Salvador and by the Research Ethical Committee (Comité de Ética de la Investigación y Bienestar Animal) of the Instituto de Salud Carlos III in Spain.

## Results

### Description of the sample

At the time of the survey 307 children and adolescents aged 2–18 years old were followed at the CENID; and under ART. Descriptive characteristics of the sample are summarized in Table 1. In nearly all the children (303/307), vertical transmission of HIV-1 was documented; in the remaining four, the mode of transmission was unknown. Mean age at the survey was 9.6 (standard deviation 0.2), with 70.4% of the children under 12 years of age. Around 70% lived in urban settings. Eleven percent of the children were institutionalised, 14% were of low-middle class and 74.9% were living in poverty or extreme poverty. Fifty-four percent of the children were on treatment for more than 5 years, and in 73.6% the viral load was undetectable.

**Table 1:** Demographic, socioeconomic and clinical characteristics of the HIV-infected children attending at CENID, El Salvador in 2011 (N = 307).

Variables	Categories	N	%
Total children		307	100.0
Sex			
	Male	161	52.4
	Female	146	47.6
Age			
	<12 years	216	70.4
	>12 years	91	29.6
Socioeconomic status			
	Institution	34	11.1
	Medium-Low	43	14.0
	Poverty	143	46.6
	Extreme poverty	87	28.3
Residence			
	Urban	215	70.0
	Rural	92	30.0
Viral load			
	<50 (copies/mL)	226	73.6
	>50 (copies/mL)	81	26.4
Time of treatment			
	<5years	138	45.9
	>5 years	163	54.2

### Nutritional status

Of the 307 children, 33.2% were stunted, 3.3% were wasted, and 10% were overweight or obese (Table 2). Stunting was more prevalent in male (37.3%) and in children above 12 years of age (48.4%). Similarly, wasting was higher in male children and more than 5 times higher in adolescents (7.7%) compared to younger children (1.4%). In contrast, overweight/obesity was more prevalent in children below 12 years of age compared to the other group (12% vs. 5.5% respectively).

**Table 2:** Prevalence of nutritional indicators by sex and age (N = 307).

	Stunted % (95% CI)	Wasted % (95% CI)	Overweighted/Obese % (95% CI)
**Sex**			
Male	37.3 (30.1–45.0)	3.7 (1.7–8.1)	11.2 (7.1–17.1)
Female	28.8 (22.0–36.7)	2.7 (1.0–7.1)	8.9 (5.2–14.8)
p-value	0.114	0.627	0.509
**Age group**			
<12 years	26.9 (21.3–33.2)	1.4 (0.5–4.3)	12.0 (8.3–17.1)
>12 years	48.4 (38.2–58.6)	7.7 (3.7–15.4)	5.5 (2.3–12.6)
p-value	0.000	0.004	0.082
**Total**	33.2 (28.2–38.7)	3.3 (1.8–6.0)	10.1 (7.2–14.0)

The association between nutritional status and the adherence to each of the dietary patterns identified by PCA was explored and no significant results were obtained.

### Food consumption

The results of the food frequency questionnaire showed that almost all children had cereals on a daily basis, and 86% were consuming legumes three or more times a week. However only 61%, 28% and 32% had a daily consumption of fruits, vegetables and dairy, respectively. Regarding animal source products, 63% of the children reported to consume eggs three times a week or more, and the consumption of fish and meat was equally divided between the “three or more times a week” (41%) and the “one-two times per week” (44%) categories. The miscellaneous group (which included sweets, sugar-sweetened drinks, sodas, sugar, pizza and chocolate drinks, among others) was consumed by 84% of the children more than three times a week, and by 60% on a daily basis. (See S1 Table).

The PCA identified three factors ordietary patterns (Table 3). Factor 1 was characterised by high factor loading for fruits, vegetables, dairy products and the flesh food group (meat and fish) and we labelled it as the “healthy diet” pattern. Factor 2 was characterised by high loading for eggs, flesh, oils and fats and the miscellaneous group, and was labelled as “high fat/sugar diet”. Factor 3 was only highly loaded by the cereals and legumes food groups and it was labelled as “low dietary diversity” dietary pattern. These patterns explained 21%, 15% and 13% of the variation in the food groups intake, respectively, and 49.4% collectively.

**Table 3:** Factor loadings for food groups in varimax rotated principal components.

	Factor 1^¶^	Factor 2^¶^	Factor 3^¶^
Eigenvalue	1.9	1.4	1.2
% Variance explained	21.1	15.0	13.4
Group 1 Cereals	0.1	0.0	0.8*
Group 2 Legumes	0.2	0.0	0.7*
Group 3 Fruits	0.7*	0.0	0.0
Group 4 Vegetables	0.8*	0.0	-0.1
Group 5 Dairy	0.7*	0.0	0.2
Group 6 Eggs	-0.1	0.6*	0.0
Group 7 Meat/Fish	0.5*	0.3*	0.1
Group 8 Oils/Fats	0.1	0.6*	-0.1
Group 9 Miscellaneous	0.1	0.8*	0.1

^¶^ Factor 1 = "healthy diet" Factor 2 = "hig fat/sugar diet" Factor 3 = low dietary diversity diet

* Food grous with factor loadings |>0.2|

**Fig 1:**
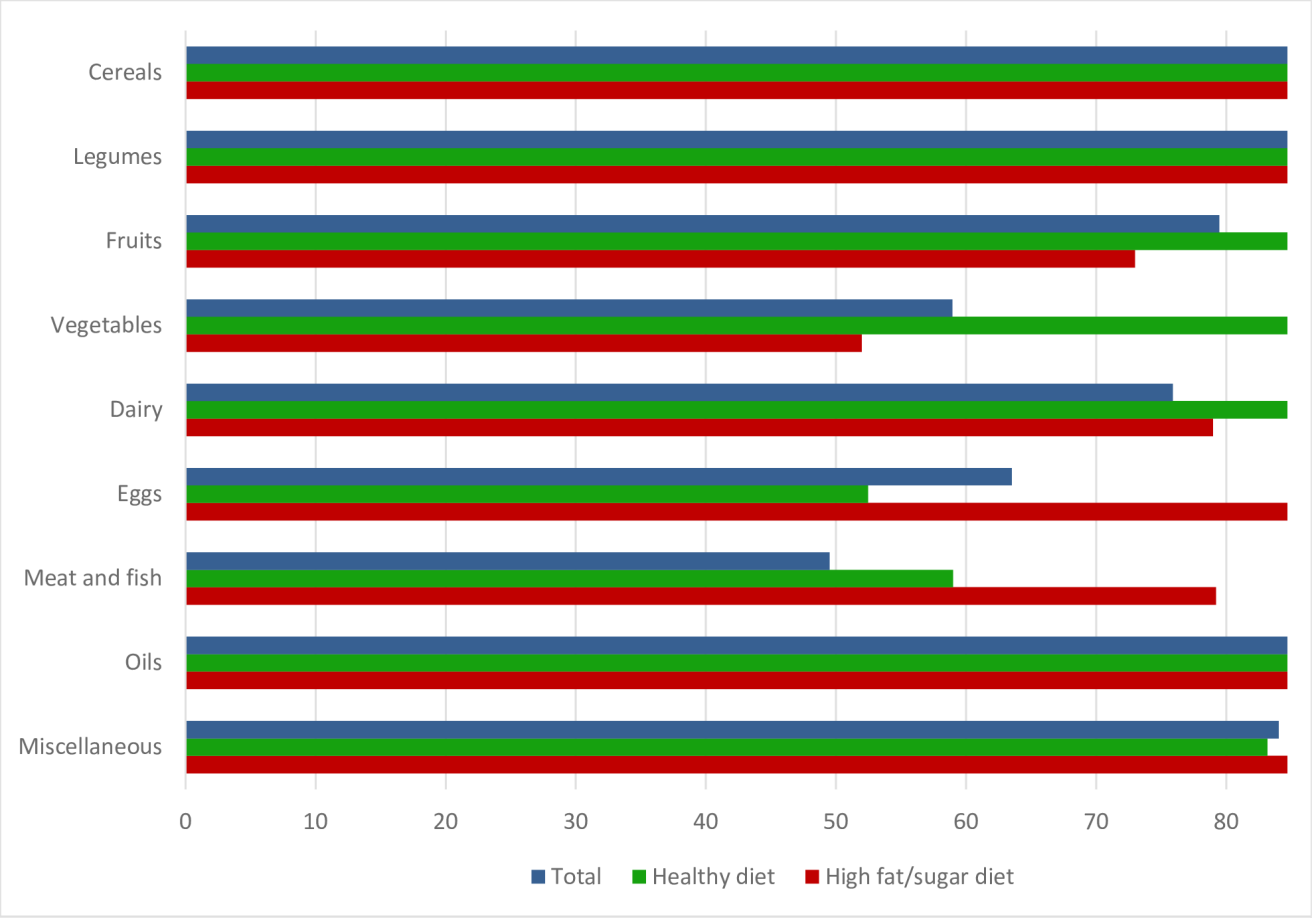
Percentage of children consuming each food group three or more times a week, by dietary pattern.

Fig 1 shows the percentage of children (for the overall population and disaggregated for children with high adherence to the “healthy diet” and the “high fat/sugar diet” patterns separately) consuming any given food group with a frequency of “three or more times a week”.

In S2 Table we summarized the food items consumed by at least 10% of the children of our sample according to the 24-hour recall, and disaggregated the analysis by high adherence to either the “healthy diet”, the “high fat/sugar diet” or the “low diversity diet”. In general, the food items more reported to have been consumed were corn tortillas, rice, beans, tomatoes, onions, cheese, eggs and sugar. Children with a high adherence to the “healthy diet” pattern consumed more meat, milk, cheese and a more diversified intake of cereals including corn tortillas, rice, pasta and maize flour in the previous day. In contrast, children with a high adherence to the “high fat/sugar diet” pattern consumed more eggs, sweets, sugar-sweetened beverages and cacao/coffee drinks. For the low dietary diversity pattern the food items consumption was not as monotonous as expected, although it must be taken into account that the dietary pattern calculation was based on the week FFQ and the food items reflects the results of the 24 hours recall.

The results of the multivariable regression analyzing the factors associated to each of the three dietary patterns are shown in Table 4. Being female, younger than 12 years, and institutionalized increased the odds to adhere to the “healthy diet” pattern, whereas living in extreme poverty decreased them.

**Table 4:** Multivariate model result for each of the dietary pattern of the HIV-infected children attending at CENID, El Salvador in 2011.

		Pattern 1 "Healthy diet"	Pattern 2 "High fat/sugar diet"	Pattern 3 "Low diversity diet"
Variables	Categories	AOR (95% CI)†	AOR (95% CI)†	AOR (95% CI)†
Sex	Male	ref	ref	ref
	Female	1.63 (0.97, 2.75)	1.14 (0.69, 1.90)	0.85 (0.52, 1.41)
*p*		0.067	0.602	0.546
Age group	>12 years	ref	ref	ref
	<12 years	2.37 (1.28, 4.36)	0.62 (0.36, 1.08)	0.83 (0.49, 1.42)
*p*		0.006	0.092	0.501
Socioeconomic status	Extreme poverty	ref	ref	ref
	Poverty	2.29 (1.17, 4.46)	1.36 (0.77, 2.39)	1.02 (0.58, 1.78)
	Institution	14.5 (5.35, 39.5)	-	0.11 (0.02, 0.48)
	Medium-low	2.65 (1.12, 6.28)	0.57 (0.24, 1.33)	0.81 (0.37, 1.78)
*p*		<0.0001	<0.0001	0.004
Residence	Urban	ref	ref	ref
	Rural	0.99 (0.55, 1.81)	1.15 (0.66, 1.98)	0.97 (0.57, 1.69)
*p*		0.997	0.628	0.94

†Adjusted Odds Ratios result of the multivariable logistic regression model including all covariates described in the tabe.

On the contrary, being older than 12 years seemed to increase the odds to adhere to the “high fat/sugar” diet (p = 0.09), as well as living in poverty or extreme poverty as compared to low-medium class children. There were no children with high adherence to this diet among the institutionalized ones.

The only variable increasing the odds of adhering to a “low dietary diversity” diet was the category of extreme poverty of the socioeconomic status.

## Discussion

The present study evaluated the nutritional status and dietary patterns of HIV-infected children under follow up at the CENID in San Salvador, El Salvador. We found a high prevalence of stunting and low wasting. Overweight prevalence was at 10%. In general, there was a low consumption of fruits, vegetables and dairy products in this population, and the “healthy diet” pattern identified was more common among institutionalized children than among children from any other socio-economic group.

More than a third of children were stunted in our study population (34.4%), a much higher estimate than the national prevalence of 14% reported in 2014 for the overall population, and the 24% estimated for the poorest quintile (which may be more similar in terms of socioeconomic characteristics to our study population, as 75% of the children were reported to in poverty or extreme poverty) [23]. Stunting was more prevalent in children older than 12 years (48.4%), which could be related to the cumulative effect of unfavorable conditions and the disease progression with age, but also to the absence and/or low quality of treatment in the first years of life of this generation of children, as the ART started to be offered free of charge by the Ministry of Health in El Salvador only in 2001. Studies in HIV-infected children in Asian and African countries have also found high stunting prevalences, ranging from 37% to 66%, depending on the context [24][25][26]. Stunting has been previously associated with HIV perinatal infection [27], and it has been described that HIV infection and the metabolic disturbances that it can induce may result in poor linear growth and weight gain [28].

The prevalence of overweight and/or obesity was higher in children below 12 years of age (12%) compared to children above 12 years (5.5%). The last Salvadorian national estimates of overweight were 6% in children under 5 years [29], and 29% in adolescents from 13 to 15 years of age [30]. Although these estimates cannot be directly compared with our results due to discrepancies in the age range of the children surveyed, we consider the prevalence of overweight in children younger than 12 years, to be high in our study population, as the prevalence for children under 5 years of age was 6% overall and 4% for the poorest quintile of the population[29].The high prevalence of stunting and overweight in this population may be reflecting the double burden of malnutrition that exists in this country undergoing nutritional transition [31] that has also been observed in other Latin American populations including non HIV-infected children from Peru [32], Mexico [33] and Guatemala [34]. This can be attributed to rapid urbanization and the adoption of diets high in refined carbohydrates and fats, combined with a more sedentary lifestyle [35]. The consequences of this double burden are exacerbated in HIV-children who, due to the disease itself, and to the treatment, are already at high risk of suffering metabolic complications. In a previous study conducted with the same sample of HIV-children of our study, it was found that high adherence to a “high fat/sugar diet” was associated with elevated risk for dyslipidemia [36].

Considering the food and nutrition recommendations of the El Salvador Ministry of Health [37] and the WHO dietary guidelines for HIV-infected children [13], the results found in relation to diet in this population are worrisome. Less than a third of the children consumed vegetables on a daily basis; around half consumed dairy products daily and more than two-thirds consumed unhealthy food items every day, which is in line with the results of the National School Survey conducted in adolescents from 13 to 15 years sampled from the overall population [30]. Similar results were also found in a study that developed and tested a household-level diet quality indicator to address the nutritional issues in 140 households from vulnerable communities in El Salvador [38]. Like us, they found excesses in eggs and sugar consumption, while the deficits came from not eating enough fruit, vegetables, meats, and dairy products. Although eggs are an important source of protein and micronutrients, its consumption is also associated with high fat/sugar diets because it is one of the main ingredients of the Salvadorian pastry and fried foods.

Only half of the children in our population consumed dairy products daily. Dairy products are essential during the growing and development periods in children and adolescents in order to attain an adequate bone mineral density and reduce the risk of osteoporosis in adult life. Several studies have found that bone abnormalities such as low bone mineral density and low bone mass accrual (particularly amongst children treated with Tenofovir), are a concern for perinatally infected children who may be at higher risk for bone fractures and osteoporosis [39] [40] [41]. In addition, there was a high consumption in our population of carbonated and low nutrient dense beverages which was found to reduce bone mineral accrual and BMC in teenage girls by replacing milk beverages in a Canadian study [42].

Daily consumption of food items from the miscellaneous group, which are in general unhealthy, is much higher than that of vegetables, dairy products, meat and fish and nearly equal to that of fruits. These results are in line with those found in the study “Análisis de la Situación Alimentaria en El Salvador” from 2011 and the 2013 National School Survey [43]. These food items have a high fat, sugar and salt content which in the long term may result in obesity and cardiovascular diseases; and their consumption usually substitutes that of healthier foods such as fruits and dairy products [42]. In our population, the intake of sodas and sweets was higher in children with a high adherence to the “high fat/sugar diet” pattern, which were more likely to live in poverty and extreme poverty. This higher consumption of sodas in lower socioeconomic strata has also been reported in Mexican adolescents [44].

The low diversity dietary pattern associated with poverty and extreme poverty identified in the study may be reflecting a diet based in maize and beans as main staples with little contribution from the other food groups as it has been described in low socioeconomic strata of other countries from the region [45]. Dietary patterns may vary depending on the cultural, geographic and economic context; however, the healthy and unhealthy or junk food dietary patterns are described repeatedly in studies from Brazil and Europe [46] [47] [48] [49]. We found different associations between having a high adherence to the “healthy diet” pattern and the socio-economic status. Children living in an institution were 14 times more likely to adhere to the “healthy diet” pattern. This might be due to the fact that the menus served in the institution were designed by a nutritionist. On the other hand, children living in poverty were the least likely to adhere to this pattern. This is consistent with a European study including 8 countries in which they found that children from lower socioeconomic status were more likely to consume processed foods, suggesting that children of poor parents could be at a higher risk of having an unhealthy diet [49]. In our study girls were more likely to adhere to the “healthy diet” pattern, and this has also been found in other studies [50].

The dietary habits of our study population seem to be in line with the dietary practices of non HIV-infected school children and adolescents in El Salvador or similar contexts, thus not conditioned by the HIV infection. This may be related to the fact that at the time of the survey all the children surveyed were asymptomatic, and that the protocols at the CENID establish to change the medical therapy in the case of prolonged gastrointestinal symptoms. Thus, disease or treatment related symptoms that could be affecting the dietary intake are scarce in this specific population. However, as the HIV infection becomes a more chronic disease, ensuring HIV-infected persons access to high quality, nutritious food choices that promote optimal dietary patterns, is of increasingly importance. A cross-sectional study, revealed that adherence to the Mediterranean diet reduced cardiovascular risk factors in a sample of 227 HIV-positive patients with the HAART-induced metabolic syndrome [51].

Moreover, optimizing the social infrastructure for the family may have a strong impact on the nutritional condition of the HIV-infected child. Caregivers should be counselled on optimum local food choices and preparation methods to ensure maximal micronutrient intake through healthy eating. Food support programmes may be beneficial. Nutrition counselling and support should aim to enable caregivers to provide a balanced diet that meets energy, protein and micronutrient needs [52].

## Limitations

The cross-sectional nature of the study does not allow examining causality in the relationship between dietary patterns and associated factors. Another limitation of the study is that data was collected during 2010–2011, and changes in this population, and in the economic, food, and health policies in El Salvador may have taken place in last years. However the last published data on dietary patterns of the El Salvador overall population dates of 2011, and this study was the first to assess dietary patterns of HIV-infected children and adolescents in the country. In relation to diet, it would have been desirable to have collected the 24-hour recall at least during 3 different days including total quantities consumed. However, logistic and financial limitations of the project did not permit to do so. In order to minimise these limitations we also collected dietary data using a weekly food frequency questionnaire, which gives information on different days than that of the 24-hour recall and offers and indirect approximation to the quantity ingested. However, FFQ are limited by possible recall bias.

## Conclusion

Stunting prevalence was high and overweight prevalence at 10% among HIV-infected children and adolescents in El Salvador. Their diets were predominantly based on a high consumption of cereals, beans, eggs and processed foods and a low consumption of fruits, vegetables and dairy products, which is in line with results from other national surveys conducted in the general population of El Salvador. These results could be reflecting the nutritional transition El Salvador is experiencing.

Institutionalized children were more likely to adhere to a healthy dietary pattern whereas children in poverty were more likely to have less varied and healthy diets.

The stunting prevalence was higher than what is estimated for the non HIV infected population from El Salvador, but no other marked differences in terms of weight for height indicators or dietary patterns were observed. However, as HIV infected children and adolescents are at higher risk of developing metabolic alterations, we highlight the need to assess their dietary practices in different context in order to advocate for interventions promoting a healthy life style as a complement to their pharmacological therapy.

## Supporting information

S1 Table Total groups in food frequency questionnaire administered to the HIV-infected children attending at CENID, El Salvador in 2011. (DOCX)

S2 Table Food groups and food items consumed by at least 10% of the HIV-infected children attended at CENID in El Salvador in 2011, by dietary pattern “high adherence”. (DOCX)
