# Supplementary material for: Dietary patterns and nutritional status of HIV-infected children and adolescents in El Salvador: A cross-sectional study
Source: PLoS One. 2018 May 15;13(5):e0196380. doi: 10.1371/journal.pone.0196380 (PMC5953455; doi:10.1371/journal.pone.0196380)
Supplement: S1 Table — (DOCX) [file pone.0196380.s001.docx]

| **S1 Table. Total groups in food frequency questionnaire (Graphs to present)** | | | | | | | | | | |
| --- | --- | --- | --- | --- | --- | --- | --- | --- | --- | --- |
|  | Daily | | 3 or more times a week | | 1 or 2 times a week | | Less than 1 a week | | Never or almost never | |
|  | n | % | n | % | n | % | n | % | n | % |
| Group 1 Cereals and tubers | 302 | 98.4 | 5 | 1.6 | 0 | 0 | 0 | 0 | 0 | 0 |
| Group 2 Legumes | 223 | 72.6 | 44 | 14.3 | 27 | 8.8 | 1 | 0.3 | 12 | 3.9 |
| Group 3 Fruits | 186 | 60.6 | 58 | 18.9 | 48 | 15.6 | 3 | 0.9 | 12 | 3.9 |
| Group 4 Vegetables | 86 | 28.0 | 95 | 30.9 | 97 | 31.6 | 9 | 2.9 | 20 | 6.5 |
| Group 5 Dairy products | 156 | 50.8 | 77 | 25.1 | 57 | 18.6 | 7 | 2.3 | 10 | 3.3 |
| Group 6 Eggs | 105 | 34.2 | 90 | 29.3 | 75 | 24.4 | 11 | 3.6 | 26 | 8.5 |
| Group 7 Meat/Fish | 26 | 8.5 | 126 | 41.0 | 135 | 44.0 | 10 | 3.3 | 10 | 3.3 |
| Group 8 Oils and fats | 284 | 92.5 | 18 | 5.8 | 5 | 1.6 | 0 | 0 | 0 | 0 |
| Group 9 Miscelaneous | 185 | 60.3 | 73 | 23.8 | 41 | 13.4 | 5 | 1.6 | 3 | 0.9 |
