# Supplementary material for: Dietary patterns and nutritional status of HIV-infected children and adolescents in El Salvador: A cross-sectional study
Source: PLoS One. 2018 May 15;13(5):e0196380. doi: 10.1371/journal.pone.0196380 (PMC5953455; doi:10.1371/journal.pone.0196380)
Supplement: S2 Table — (DOCX) [file pone.0196380.s002.docx]

| **S2 Table. Food groups and food items consumed by at least 10% of the HIV-infected children attended at CENID in El Salvador in 2011, by dietary pattern “high adherence”** | | | |
| --- | --- | --- | --- |
| **Food groups and food items** | **"Healthy diet"** | **"High fat/sugar diet"** | **“Low diversity diet”** |
|  | **(n=101)** | **(n=100)** | **(n=102)** |
|  | **%** | **%** | **%** |
| Group 1. Cereals y tubers |  |  |  |
| Rice | 80.2 | 54.0 | 45.1 |
| Maize | 28.7 | 13.0 | 11.8 |
| Bread | 53.5 | 58.0 | 56.9 |
| Potato | 21.8 | 26.0 | 27.5 |
| Banana | 23.8 | 23.0 | 15.7 |
| Pasta | 27.7 | 13.0 | 13.7 |
| Corn tortilla | 90.1 | 92.0 | 93.1 |
| Maize flour | 19.8 | 1.0 | 1 |
| Group 2. Legumes |  |  |  |
| Beans | 79.2 | 78.0 | 83.3 |
| Soy | 24.75 | 3.0 | 3.9 |
| Group 3. Fruits |  |  |  |
| Guineo | 20.8 | 15.0 | 21.6 |
| Orange | 14.9 | 18.0 | 10.8 |
| Mango | 7.92 | 13.0 | 16.7 |
| Jugo natural | 31.7 | 25.0 | 20.6 |
| Apple | 14.85 | 6.0 | 5.9 |
| Group 4. Vegetables |  |  |  |
| Tomato | 43.6 | 48.0 | 50 |
| Onion | 33.7 | 34.0 | 37.3 |
| Chile | 28.7 | 28.0 | 30.4 |
| Carrot | 14.9 | 12.0 | 10.8 |
| Güisquil | 10.9 | 13.0 | 9.8 |
| Cabbage | 21.8 | 5.0 | 0 |
| Group 5. Dairy products |  |  |  |
| Cheese | 63.4 | 48.0 | 56.9 |
| Milk | 71.3 | 38.0 | 39.2 |
| Cream | 30.7 | 41.0 | 27.5 |
| Group 6. Eggs |  |  |  |
| 37.6 | 67.0 | 56.9 |  |
| Group 7. Meat and fish |  |  |  |
| Poultry | 32.7 | 28.0 | 30.4 |
| Meat | 41.6 | 19.0 | 14.7 |
| Sausages | 29.7 | 12.0 | 11.8 |
| Cold meat | 8.9 | 18.0 | 13.7 |
| Fish | 5 | 3.0 | 9.8 |
| Group 8. Oils and fats |  |  |  |
| Oil | 11.9 | 5.0 | 12.8 |
| Group 9. Miscellaneous |  |  |  |
| Sugar | 80.2 | 84.0 | 81.4 |
| Coffee and cacao beverages | 38.6 | 74.0 | 64.7 |
| Sweets | 44.6 | 72.0 | 60.8 |
| Sodas | 37.6 | 57.0 | 46.1 |
| Sugar sweetened beverages | 29.7 | 10.0 | 8.8 |
